# PCR-Activated Cell Sorting for Cultivation-Free Enrichment and Sequencing of Rare Microbes

**DOI:** 10.1371/journal.pone.0113549

**Published:** 2015-01-28

**Authors:** Shaun W. Lim, Tuan M. Tran, Adam R. Abate

**Affiliations:** 1 UC Berkeley-UCSF Graduate Program in Bioengineering, University of California San Francisco, San Francisco, California, United States of America; 2 Department of Bioengineering and Therapeutic Sciences, California Institute for Quantitative Biosciences, University of California San Francisco, San Francisco, California, United States of America; Texas A&M University, UNITED STATES

## Abstract

Microbial systems often exhibit staggering diversity, making the study of rare, interesting species challenging. For example, metagenomic analyses of mixed-cell populations are often dominated by the sequences of the most abundant organisms, while those of rare microbes are detected only at low levels, if at all. To overcome this, selective cultivation or fluorescence-activated cell sorting (FACS) can be used to enrich for the target species prior to sequence analysis; however, since most microbes cannot be grown in the lab, cultivation strategies often fail, while cell sorting requires techniques to uniquely label the cell type of interest, which is often not possible with uncultivable microbes. Here, we introduce a culture-independent strategy for sorting microbial cells based on genomic content, which we term PCR-activated cell sorting (PACS). This technology, which utilizes the power of droplet-based microfluidics, is similar to FACS in that it uses a fluorescent signal to uniquely identify and sort target species. However, PACS differs importantly from FACS in that the signal is generated by performing PCR assays on the cells in microfluidic droplets, allowing target cells to be identified with high specificity with suitable design of PCR primers and TaqMan probes. The PACS assay is general, requires minimal optimization and, unlike antibody methods, can be developed without access to microbial antigens. Compared to non-specific methods in which cells are sorted based on size, granularity, or the ability to take up dye, PACS enables genetic sequence-specific sorting and recovery of the cell genomes. In addition to sorting microbes, PACS can be applied to eukaryotic cells, viruses, and naked nucleic acids.

## Introduction

Microbial communities play crucial roles in geochemistry and exist in diverse ecosystems[1,2]. Understanding the genetics of the cells that inhabit an ecosystem is critical to understanding how microbes function individually and in the complex networks that make up the natural environment [1,2]. Studying the genetics of individual microbes, however, is difficult because most cannot be cultured in the laboratory and comprise uncultivable “microbial dark matter”[3,4]. To study uncultivable microbes, cultivation-free methods like shotgun sequencing are necessary. In this approach, nucleic acids are purified out of a heterogeneous sample via chemical means, sheared into short fragments, and sequenced. To assemble the resulting compilation of short sequences into a larger coherent dataset, computational algorithms are utilized, but this process is often hampered by the lack of sequencing depth and the complexity of the diverse set of sequences obtained [5–7]. As a result, next-generation sequencing of diverse communities commonly yields information about the genes present in a system but is unable to tell how those genes are bundled into genomes and packaged into individual cells. The inability to correlate sequences present within a single microbe prevents the association of distinct biosynthetic pathways that interact to form important phenotypes that can impact the global ecology of the system. Moreover, in such analyses, the genes of rare microbes are difficult to detect since they tend to be swamped out by the sequences of the off-target microbes that greatly outnumber them [7,8]. This makes studying low-abundance microbes with interesting phenotypes particularly difficult.

One strategy for obtaining the genomic sequences of rare microbes in a diverse population is target enrichment. In this approach, fragments of the genomes of the target microbes are recovered by hybridization to capture probes. Sequence complementarity between the probes and targets allows the molecules to anneal, so that the target fragments can be recovered via probe enrichment [9,10]. A limitation of probe capture, however, is that recovering whole microbial genomes requires hundreds or thousands of overlapping capture probes [11], necessitating substantial knowledge of the target sequence, which may not be available. Moreover, even when capture probes can be designed, the fragments captured are limited to those near the sequences targeted by the probes, biasing what can be detected by what is already known; this precludes recovery of whole genomes in many instances and, thus, prevents complete genetic characterization of the species of interest. This is particularly problematic when horizontal transfer of genetic elements occurs because, in such unpredictable instances, these sequences are not known to exist in the species of interest and, thus, it is not possible to construct probes with which to capture them. Horizontal gene transfer is an important method by which microbes transfer genetic information and generate phenotypic diversity [12], which is why detecting such events is essential for increasing our understanding of microbe evolution.

To overcome the limitations of probe hybridization capture, a superior method would be to label the target microbes with a specific reporter; the labeled cells could then be recovered, together with their whole genome, using ultrahigh-throughput fluorescence-activated cell sorting (FACS). One method for accomplishing this is to chemically fix and permeabilize microbes and then bind to their nucleic acids probes labeled with fluorescent dyes; the then fluorescent cells can be sorted with FACS, a method known as fluorescence in-situ hybridization, fluorescence-activated cell sorting (FISH-FACS) [13]. FISH-FACS has enormous benefits over probe hybridization capture because it allows cultivation-free enrichment of the whole genome of the microbe of interest. However, FISH-FACS also has drawbacks that significantly limit its applicability for sequencing microbes. For example, fixation can chemically modify DNA and introduce sequencing bias and errors into the genomes recovered, yielding poor sequence data [14,15]. More importantly, achieving bright, specific labeling of the cell type of interest requires substantial trial-and-error optimization of the fixation and permeabilization procedure, something that is not possible when seeking to recover the genome of a cell that cannot be cultured in the lab. This is challenging when screening natural samples containing large numbers of different microbes with distinct cell wall and membrane properties[16]. Consequently, while FISH-FACS holds enormous utility for the *in situ* identification of nucleic acid sequences in uncultivable microbes, it has drawbacks which limit its routine use for sequencing purposes. To enable the robust whole-genome sequencing of rare, uncultivable microbes, a new method for enriching intact microbial genomes out of a diverse ecosystem is needed.

In this paper, we introduce PCR-Activated Cell Sorting (PACS) for the cultivation-free enrichment of rare microbial genomes. In PACS, microbes from a diverse ecosystem are individually encapsulated in picoliter-volume aqueous droplets and subjected to TaqMan PCR, interrogating them for the presence of specific nucleic acid sequences. If the sequences are present, TaqMan amplification yields a bright fluorescent signal that fills the droplet encapsulating the cell, allowing us to recover the cell’s whole genome by sorting the droplet. PACS has a number of advantages for recovering rare microbial genomes. Because it utilizes PCR to identify the microbes of interest, harsh lysis procedures that include temperatures near the boiling point of water can be used, reducing erroneous identification compared to methods that rely on room-temperature probe hybridization. In addition, because these reactions are performed on single cells using ultrahigh-throughput microfluidics, millions of cells can be screened in hours, making the approach well adapted for recovering rare microbial genomes in a large and diverse sample. PACS thus realizes the potential of FISH-FACS for targeted metagenomics while being more robust and simpler to implement, since it does not require fixation of the cells and, rather than relying on room-temperature probe hybridization, relies on robust, easy to optimize, and straightforward to target TaqMan PCR.

## Results and Discussion

### The strategy of PACS

The motivating concept of PACS is to enable ultrahigh-throughput sorting of cells based on the sequences contained within their genome. We accomplish this by utilizing TaqMan PCR, which has known advantages of sensitivity and specificity. Upon exponential amplification of target DNA from single-copy genomic material, a bright signal is generated in the droplets containing the target cells, allowing robust discrimination and recovery of these cells within a large population of off-target cells. Additionally, TaqMan PCR enables specific identification of microbial genotypes by allowing for multiplexing, which enables simultaneous interrogation of several genomic regions in the same microbe.

The benefits of PCR have been recognized previously and implemented into microchamber devices for single cell analysis. It has enabled the amplification of two distinct sequences from individual microbes for sequencing [17], and the association of specific viruses with their bacterial hosts [18]. A limitation of microchamber methods, however, is that the number of single cell reactions that can be performed is limited by the number of microchambers that can be fabricated. In addition, in microchamber methods, recovering the genomes of target microbes requires individually accessing the positive chambers using tedious micromanipulation techniques [17,18], limiting the ability of the method for screening and recovering large numbers of cells.

In PACS, we utilize microfluidic droplets in place of microchambers. Compared to microchambers, droplets are more scalable and enable the individual PCR analysis and sorting of many more cells. This is because, with microfluidic devices, droplets can be generated and sorted at kilohertz rates and each droplet utilizes just tens of picoliters of reagent, allowing millions of PCR reactions on single cells with microliters of total reagent [19]. Moreover, because the aqueous droplets are suspended in an inert liquid oil, they can be flowed through microfluidic devices, which allows multiple steps of processing, such as sampling fluid from, adding reagents to, and incubating and sorting the droplets; this allows multistep reactions in the droplets not possible in sealed microchambers [19–21].

In PACS, small genomic regions hundreds of bases in length serve as “sequence biomarkers” to identify cells of interest. Based on the PCR signal produced when a cell containing the sequence biomarker is present, droplet sorting is used to recover the entire genome of the cell. This sorting can be accomplished microfluidically, as we demonstrate here, or using double emulsification and FACS [22], which provides the benefit of allowing single genomes to be dispensed into individual wells.

In contrast to conventional antibody labeling methods and FACS sorting, PACS is easier to implement and quicker to optimize, because the detection of the microbe depends on the efficiency of robust PCR reactions rather than on antibody-epitope binding. Moreover, PACS is more easily targeted at different identifying biomarkers, since this requires only the design of different probe sequences, compared to the development of a new antibody. Another benefit of PACS is that it can utilize either PCR or RT-PCR [23,24] to analyze cell DNA or RNA; this allows differentiation between cells merely containing a pathway in their genome and those that are actively expressing it. Similarly, because PACS derives its sensitivity from PCR, it is capable of detecting individual copies of the target sequence in the cell, making it applicable to sorting based on sequences in the genome and transcriptome, or even plasmid or viral sequences.

### Targeted recovery of microbial genomes with PACS

**Fig 1:**
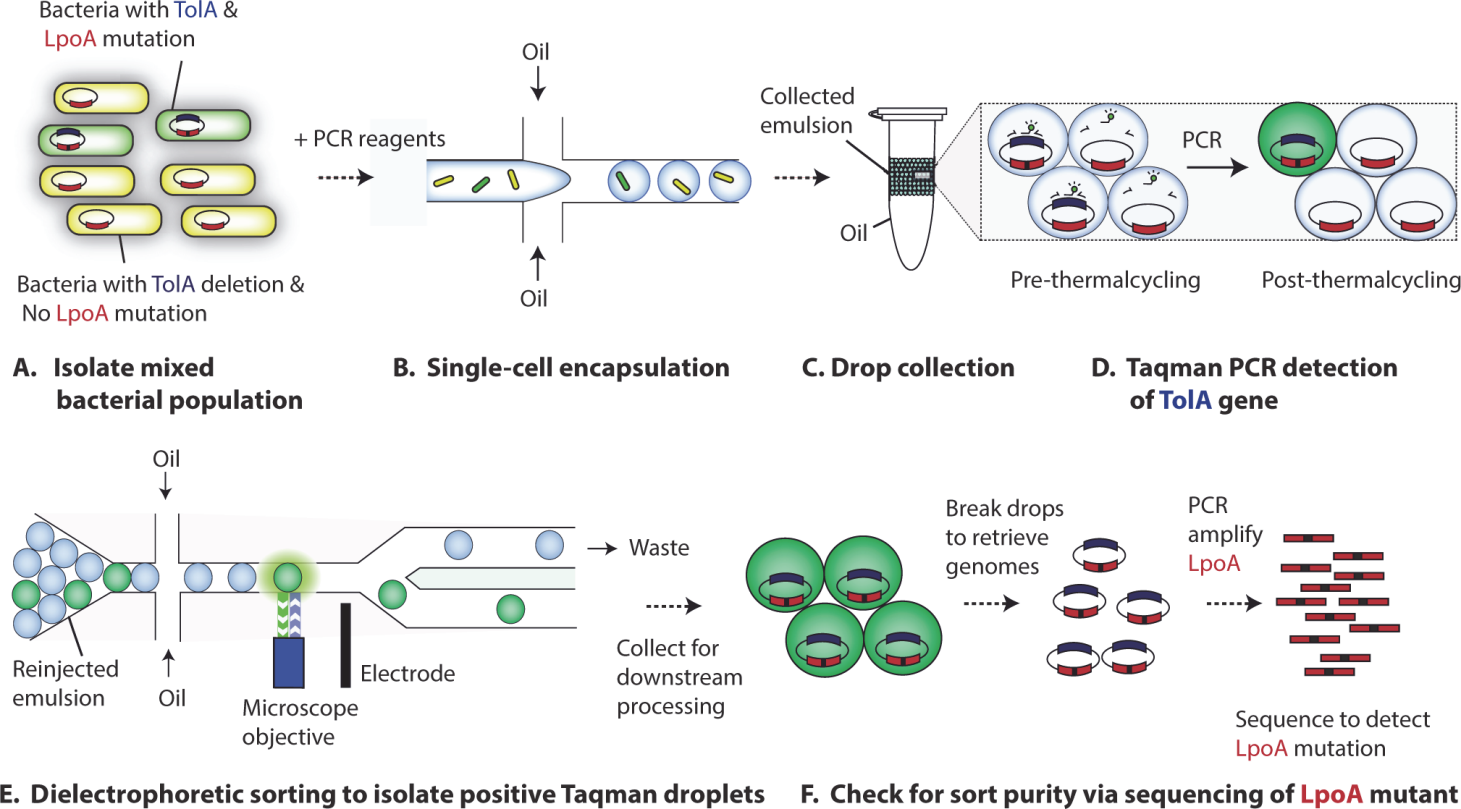
The PACS workflow applied to a model microbial system. A microbial sample consisting of K-12 E coli harboring wild type TolA and a spike-in variant (ΔTolA) is created from growth cultures (Fig. 1A). This sample is then encapsulated together with PCR reagent to form a single emulsion (Fig. 1B). This emulsion is then collected and thermocycled, with PCR-positive droplets experiencing an increase in Taqman fluorescence (Fig. 1C). This emulsion is then DEP sorted for bright drops (Fig. 1D), and these drops are ruptured to release genomic content which is sequenced to verify sorting efficacy (Fig. 1E).

The first step in PACS is to encapsulate the microbes (Fig. 1A) in individual water-in-oil droplets using microfluidic emulsification (Fig. 1B); here, we include the PCR reagent in the microbial suspension, although the microbes and PCR reagent, which can include detergents to enhance lysis, can also be combined on-chip via laminar co-flow followed by droplet generation. The microbes are diluted so that there is on average less than one per droplet, loading the droplets randomly in accordance with a Poisson distribution. The droplets are ∼35 pL in volume, although this can be varied over >5X up or down, and are collected into a PCR tube and thermocycled (Fig. 1C). We perform the thermocycling on a standard PCR machine, although on-chip thermocyclers could also be used for an unbroken workflow [25]. During PCR, the elevated temperature lyses the microbes and disrupts DNA-protein and DNA-DNA interactions, providing the PCR primers with access to the cell’s DNA. Droplets containing the genetic sequences being assayed for will result in TaqMan PCR amplification, yielding a droplet that is bright with fluorescence at the emission wavelength of the TaqMan probe due to its degradation by the 5’ exonuclease activity of Taq polymerase [26]. At this point in the process, we have millions of droplets, some of which are now fluorescent because they contain a microbe with the sequence targeted by our assay. The next step is to screen these droplets using ultrahigh-throughput dropometry and recover the positives with dielectrophoretic (DEP) sorting (Fig. 1D) [27]. The sorted droplets can be loaded into individual wells or pooled together and chemically ruptured to access their contents, providing us with the genomic DNA of the target microbes. Amplicons mixed together with the sorted genomes can be removed by a variety of methods, such as Uracil-DNA Glycosylase enzymatic digestion, if using a PCR mix with uracil, or with streptavidin beads that can extract biotin-labelled amplicons. Downstream transcriptomic analysis might necessitate the development of lysis and isothermal PCR methods that do not require high heat, as RNA is known to be heat-sensitive.

### Validation of PACS

To validate that PACS can recover specific microbes out of a mixed population, we spike target cells into a background of non-target cells. We use two different E. coli strains that have two differences in their genome: The first strain has the genetic sequence for the membrane protein TolA knocked out (ΔTolA), whereas the second has TolA intact but is a double mutant on the LpoA gene, which is an outer membrane lipoprotein (LpoA K168A) [28]. The mixed population is then run through our PACS workflow (Fig. 1) sorting based on the presence of TolA, which should only recover the LpoA double mutants. To characterize the efficiency of the PACS sorting, we recover the genomic DNA of the sorted microbes and PCR-amplify and sequence the portion of the LpoA gene containing the mutations (Fig. 1E). By comparing the number of sequences containing the double mutant and those absent of it, we are able to estimate the efficiency with which PACS can discriminate between these cell types based on TolA. That is, the objective of this experiment is to show that we can differentiate between cells based on the presence of a gene (TolA) at one location of the genome, and then confirm correct sorting by analyzing a different gene (LpoA) far away on the same genome. Importantly, the sequences analyzed post-sorting are not the product of the first PCR; they are present in the sorted mixture only because they existed in the same genome that contained TolA and, thus, were sorted with it.

### Efficiency of single cell droplet PCR

**Fig 2:**
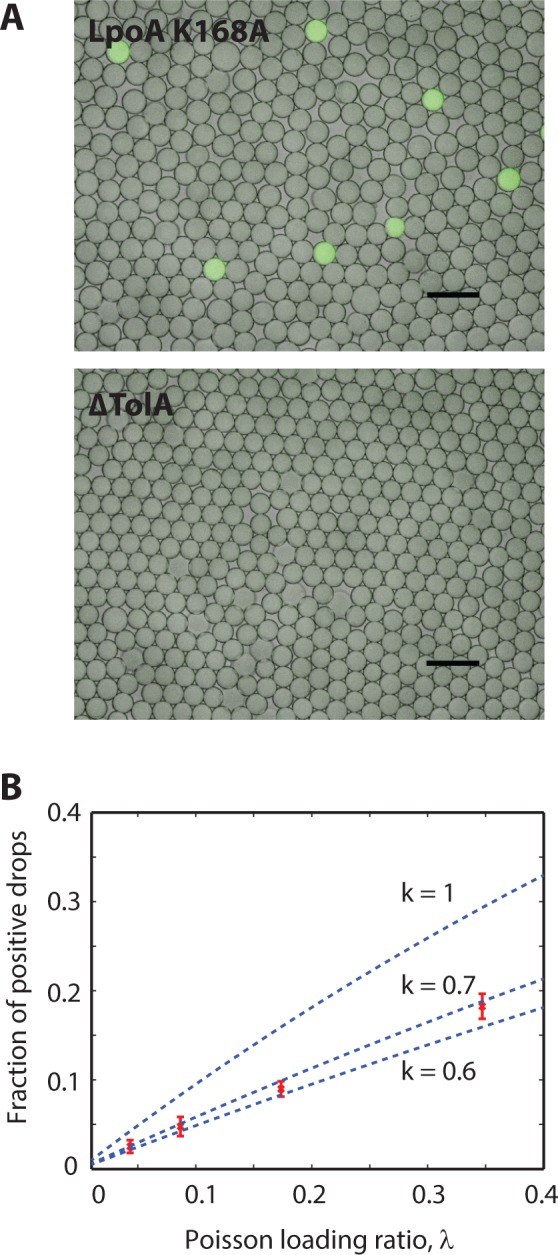
Taqman PCR detection of TolA gene on E. coli bacteria. E coli bacteria are encapsulated with PCR reagents in droplets and are thermocycled. Fig. 2A, *upper*, Drops containing bacteria with the TolA gene are bright, whereas this is absent in Fig. 2A, *lower*, which has E coli without this gene. Fig. 2B shows the dependency of the fraction of loading drops which are bright versus the poisson loading ratio. The different curves represent different calculated curves if the E. coli lysis factor *k* was varied.

The ability to PACS sort the microbes is thus contingent on the ability to specifically discriminate between the two cell types with our TaqMan assay. To investigate the specificity of the TaqMan assay, we perform control experiments in which we emulsify clonal populations of the two cell types separately, and then analyze the cells using droplet single cell TaqMan PCR with primers and probes for the TolA gene, as shown in Fig. 2A. For the droplets containing the double mutants (LpoA K168A), in which TolA is present, we observe a “digital” fluorescence signal, in which a small fraction of the droplets are bright, and the remainder exhibit no fluorescence, as illustrated in Fig. 2A, *upper*; the fluorescent droplets contain individual K168A microbes, while the dim droplets are devoid of any cells and thus constitute what we expect to see when the target sequence (TolA) is not present within the droplet. To confirm this, we perform the same experiment with the knockout population (ΔTolA), the results of which are shown in Fig. 2A, *lower*. As expected, even though the stoichiometry of the ΔTolA cells is comparable to that of the K168A cells in the first experiment, so that we expect similar loading rates into the droplets, we observe no fluorescent droplets; this demonstrates that our TaqMan assay is specific to cells that have sequences targeted by our primers. This is consistent with control experiments performed in bulk on large numbers of the cells and also with the properties of TaqMan PCR. To validate that, indeed, the positive droplets in the K168A experiment correspond to “digital” amplification resulting from a TolA positive cell, we repeat the experiment for different concentrations of K168A cells. For Poisson loading of the cells in droplets, the probability that a given droplet has *x* cells is given by,

$$P(x;\lambda )=\frac{e^{-\lambda }\lambda^{x}}{x!}$$ (1)

where *λ* is the average number of cells per 35 pL droplet (S1 Fig.). Bright drops correspond to *x* ≥ 1, whereas *x* = 0 relates to dark drops. The proportion *p* of bright to dark drops depends on *λ* according to,

$$p=1-e^{-\lambda }$$ (2)

This is a simple statement that as the concentration of cells in suspension increases, more of the droplets contain at least one cell. To relate the number of cells in the droplets to the number of fluorescent droplets observed at the conclusion of the assay, we must account for the fact that not all droplets containing single cells undergo amplification. That is, due to inefficiencies in the PCR, the probability that the reaction undergoes amplification is less than unity. We can account for this by rewriting the equation as

$$p=1-e^{-k\lambda }$$ (3)

where *k* is the probability that a droplet containing a target cell yields a fluorescent signal. To measure, *k*, an important parameter that describes the sensitivity with which we detect positive cells, we repeat the experiment at different concentrations, Fig. 2B. For *k* = 1, the TaqMan reaction can be said to be perfectly efficient so that every drop containing a cell yields a fluorescent signal. For *k* < 1, the reaction is imperfect so that some droplets containing positive cells do not yield a fluorescent signal. Based on our data, we determine 0.6 < *k* < 0.7, indicating that we detect approximately 65% of the positive cells in the sample. This inefficiency may be a consequence of the natural stochasticity of PCR, particularly in picoliter volumes in which reagents may be limiting. Another explanation is that cell lysis is not perfectly efficient, as heat lysis might not be sufficient to lyse all the bacteria. This effect can be mitigated by including PCR-compatible detergents in the droplets, which aid cell lysis and solubilization of DNA targets and may improve single cell PCR efficiency. Using more sophisticated multistep microfluidic techniques, it is also possible to include PCR incompatible lysis reagents, such as alkaline buffers, lysozyme, or proteases, to enable lysis of particularly durable microbes. Further development of lysis cocktails compatible with our workflows will be essential towards the goal of unbiased lysis of unknown microbes in environmental samples.

### Recovery of whole bacterial genomes with droplet sorting

**Fig 3:**
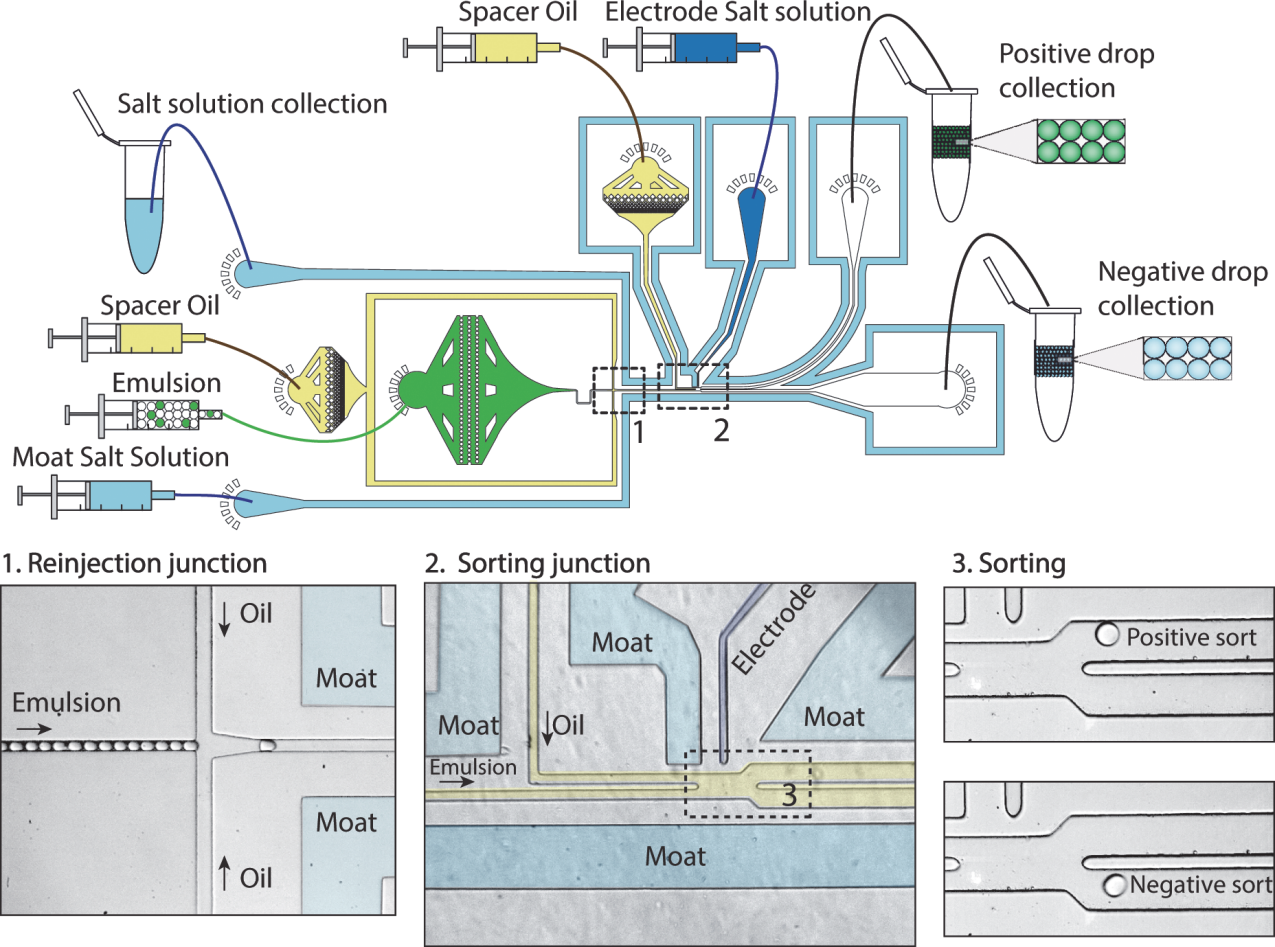
DEP droplet sorting device. Fig. 3, *upper*, shows the device layout, with the salt “moat” insulating the drops from any stray electric fields potentially originating from the salt electrode. This device consists of the reinjection junction, Fig. 3, *left*, at which the reinjected emulsion is spaced out, as well as the sorting junction, Fig. 3, *middle*, which is where detection and sorting occurs. Fig. 3, *right*, shows positive and negative droplet sorting events.

At the conclusion of our single cell droplet PCR, we have a collection of millions of droplets, some of which contain target microbes and are fluorescent. To recover the positive droplets and the genomes of the cells they contain, we use ultrahigh-throughput dielectrophoretic (DEP) droplet sorting. DEP droplet sorting has previously been employed to robustly sort droplets at several kilohertz for applications like enzyme evolution [29] and the detection of high-affinity antibodies [30]. Our droplet sorter consists of a droplet reinjection inlet, a spacing inlet, and a sorting junction. The device is surrounded by conducting aqueous “moats” that shield the injected droplets from stray electric fields, which can unintentionally coalesce droplets. These “moats” are designed so that they surround the oil channels, as illustrated in Fig. 3.

**Fig 4:**
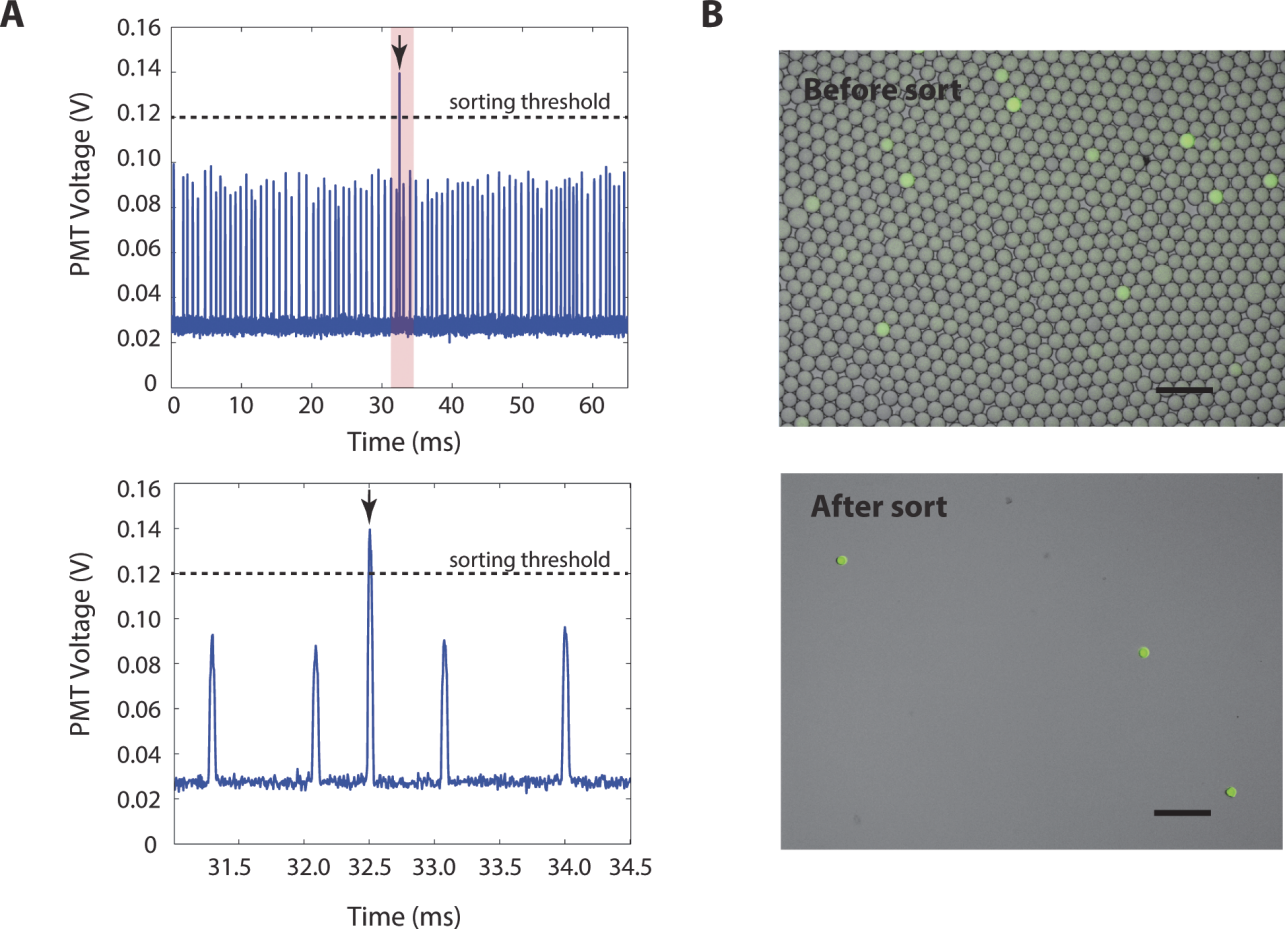
Droplet detection and sorted drops. Fig. 4, *left*, is the PMT timetrace of recorded signals from the optical droplet detection setup. There is a clear peak at 32.5 ms, which corresponds to a bright drop that is sorted. Fig. 4, *right*, are the fluorescence images of thermocycled drops before and after DEP sorting. Scale bars are 100 μm.

Upon injection into the device, the thermocycled droplets are close packed and spaced by oil in the spacing junction, as shown in Fig. 3, *left*. Spacing ensures that the droplets pass the detection region (Fig. 3, *middle*) one at a time, so that the fluorescence of each droplet can be measured individually. It also ensures that the droplets do not crowd the sorting junction (Fig. 3A, *right*), which can result in droplet collisions that interfere with controlled sorting. After spacing, the droplets pass through the detection region (Fig. 3, *middle*) and pass through a focused laser beam; the laser excites the fluorescent dyes in the droplets, causing them to emit light in proportion to the amount of cleaved TaqMan probes they contain. Droplets that underwent successful TaqMan amplification emit bright fluorescent light, while those that did not appear dim. The fluorescent light is captured by the objective of a microscope, filtered through dichroic mirrors and bandpass filters, and focused onto the sensor of a photomultiplier tube (PMT). The PMT outputs a voltage proportional to the intensity of the fluorescent light. The oil surrounding each droplet is not fluorescent; hence, when a droplet passes through the detection laser, the PMT records a peak as a function of time, as shown in Fig. 4A; each peak in the time trace corresponds to a distinct droplet. The amplitude of a given peak is proportional to the intensity of the droplet, allowing bright TaqMan positive droplets to be differentiated from dim TaqMan negative droplets, as illustrated by the bright droplet at t = 32.5 ms.

To recover the bright droplets, we set a threshold voltage to 0.12V; this value varies between runs depending on the focusing optics and PMT gains and is selected in this experiment to cleanly distinguish between positive and negative droplets, as shown in Fig. 4A. Above this value, the computer is instructed to sort the droplet, which it does by outputting an alternating current (AC) pulse that is amplified to ∼1500 V and applied to a conducting aqueous electrode in the sorting junction, as illustrated in Fig. 3, *middle*. Energizing the electrode generates an electric field that polarizes the droplet in the sorting junction; this produces a dielectrophoretic attraction that pulls the droplet towards the electrode [31], deflecting it into streamlines that carry it into the collection channel. When the electrode is not energized, the geometry of the sorting junction is designed so that the droplet follows streamlines that carry it into the waste channel. Hence, by selectively energizing the electrodes based on the measured fluorescence of the droplets, we are able to recover the TaqMan positive droplets and discard the negative droplets, as can be seen by comparing the images in Fig. 4B. Other sorting methodologies such as valve-based sorting [32], surface acoustic waves [33], and double emulsion FACS [22] could also be used to recover the positive droplets.

### Sequence verification of sorted genomes

**Fig 5:**
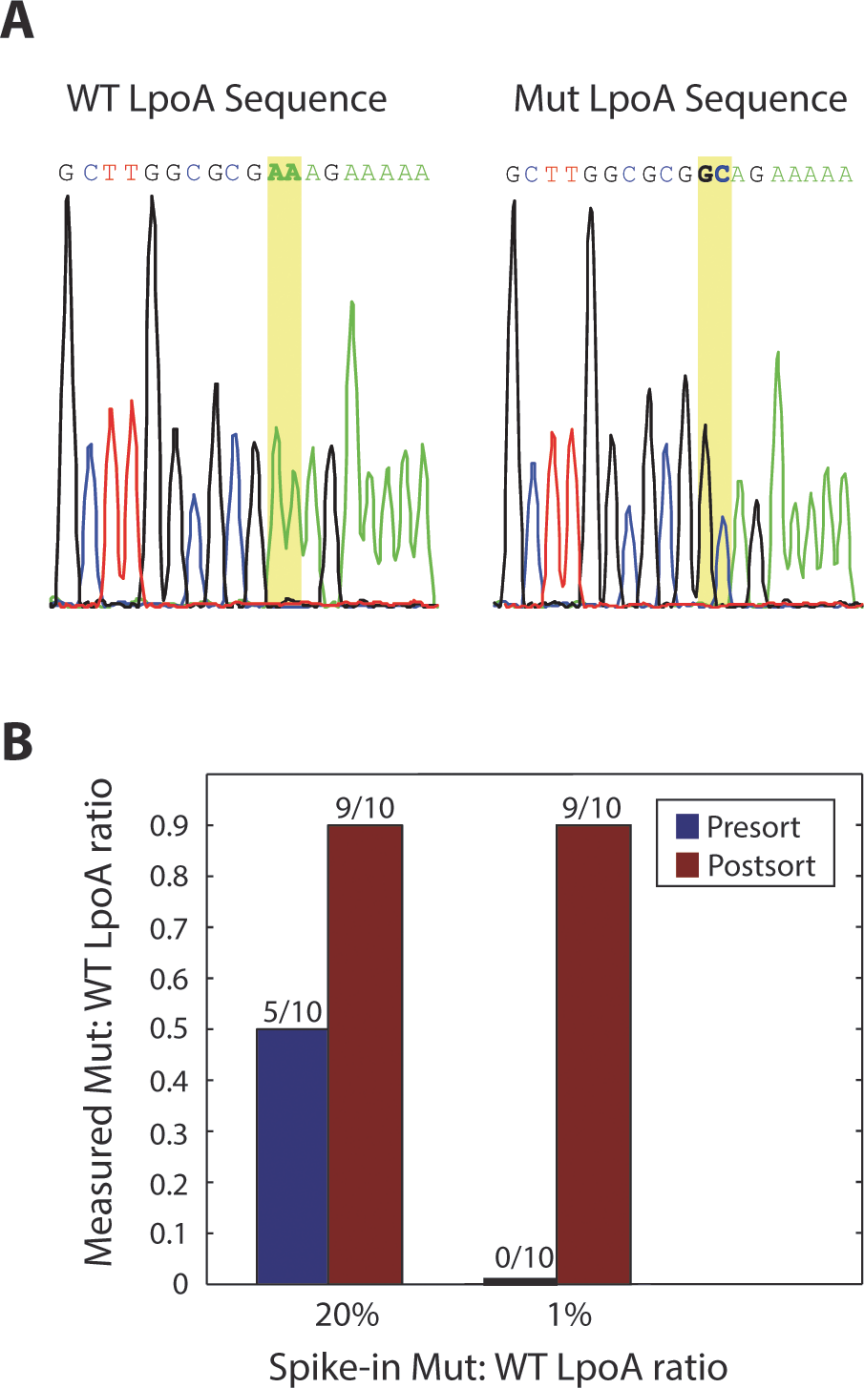
Sequencing verification and genome enrichment. Fig. 5A is a representative electropherogram of the LpoA gene and its mutant counterpart after Sanger sequencing LpoA from sorted bacterial genomes. There are clear base calls on the double nucleotide mutation. Fig. 5B shows the sequencing results, with clear enrichments for the TolA/ΔLpoA bacterial strain for both spike-in ratios.

Epifluorescence microscopy images like those of Fig. 4B demonstrate that the dielectrophoretic sorter is capable of accurately sorting the bright from the dim drops. To further validate that PACS enables accurate single cell sorting based on nucleic acid sequences, we recover the genomic DNA from the positively sorted droplets for Sanger sequencing. The sorted droplets are chemically ruptured with the addition of chloroform and application of mechanical shear, and the microbial genomes dispersed into aqueous buffer. The K168A cell line has a double amino acid mutation of “AAA” encoding lysine to “GCA” encoding alanine, as seen in Fig. 5A. Even if there are errors in the PCR preparation or the Sanger sequencing, these are expected to be rare and, extremely so, for 2 consecutive nucleotides, providing a high confidence read out with which to validate the PACS sorting. We tested two mix ratios of the ΔTolA and TolA bacteria, one where the mutant was present at 20% in the total population, and the other at 1%. For the 20% spike-in, 5 of 10 sequences before PACS were positive for the mutant, whereas 9 of 10 were positive after PACS, as shown in Fig. 5A. The high pre-PACS frequency of the mutant is likely the result of random sampling variation, since we sequenced only ten molecules. Similarly, the 1% spike-in yielded no pre-PACS positives in our ten molecule sample, while the post-PACS library was again 9 of 10. Thus, for both spike-in ratios, we observe a reasonable number of mutants pre-PACS and very few mutants post-PACS. For these two samples, the droplets were sorted at 1 kHz for 1 hour each, and at a cell loading ratio of around 0.1 cells per droplet. This translates to 3.6 million drops put through the sorter, with around 360,000 cells processed per sequencing run.

The deviation from 100% mutant sequence post-PACS may result from multiple sources. An unlikely source is library preparation or sequencing errors, since these should occur very rarely. Another possibility is erroneous sorting of the droplets. This can be due to malfunction of the sorting device, which is also rare for the conditions used or, more likely, from droplets coalescing during the thermocycling. Unintended droplet coalescence allows the contents of multiple droplets to mix together, so that a droplet with a positive signal can have added to it, by way of merger, the genomic DNA of a negative cell, thereby resulting in contamination of the sorted material. While we do observe some merger during thermocycling, it is also rare. The most likely source of the error is multiple encapsulations while loading the cells into the droplets. Multiple encapsulations can occur for several reasons, such as the cells clumping together or two or more cells entering into the droplet generation junction at the same time. Multiple encapsulations are the primary events that limit our sorting accuracy, although they can be mitigated by handling cells carefully, filtering cells to remove clumps, and diluting the suspension to limit double encapsulations. In any case, even with the error, we are able to obtain an enrichment ratio of 0.90/0.01 = 90X with one sort for the 1% spike in. If this enrichment is not sufficient, the system can be further diluted or additional rounds of PACS used to obtain high purity genomes.

## Conclusion

PACS is a new cell sorting technology that has great utility for sorting microbes out of heterogeneous populations without the need for prior cultivation. PACS derives this power from its ability to directly sort cells off of sequence-specific nucleic acids; this has advantages over antibody labeling when the cell type is not known to express a unique surface marker. PACS preserves the genomic information from individual cells allowing them to be analyzed with downstream methods like next generation sequencing. This method can be used to search microbial “dark matter” for bacteria with certain postulated metabolisms, for example chemolithoautotrophy in the ocean. We have demonstrated PACS with bacterial cell genomes and believe that this method can also be applied to analysis of cellular transcriptomes in the future, which would open up opportunities for studying single-cell gene expression in complex ecosystems.

By utilizing droplet-based mammalian cell PCR methods, PACS can be applied to human health applications, such as, detecting and analyzing circulating tumor cells or characterizing heterogeneity in cultured stem cells and tumor cell populations. PACS should also enable direct sorting of viruses or naked DNA, capabilities that have never been available in the fields of virology and genomics. For example, this should enable populations of viruses and cells to be screened for metagenomic analysis or to detect the hosts of uncultivable viruses. We expect that PACS will impact genomics by allowing large fragment enrichment. By combining PACS with recently-developed methods for sorting microfluidic droplets using flow cytometry, PACS can also be used to load individual cells, viruses, and DNA molecules into microwells for whole-genome amplification and sequencing; this should aid single cell analysis and genetic haplotyping efforts.

## Materials and Methods

### Microfabrication of devices

Fluidic chips are fabricated using standard photolithography techniques in poly(dimethylsiloxane) (PDMS) [34]. To produce a master, we first spin a layer of SU-8 photoresist (Microchem) onto a silicon wafer, and then expose the photoresist to UV light from a Blakray device under a mylar mask(Fineline Imaging). The wafer is then baked at 95^o^C on a hotplate for 1 min and then developed in Propylene glycol monomethyl ether acetate (PGMEA). We then pour PDMS polymer and crosslinker mixed in a 11:1 ratio over the master and then bake it at 75^o^C for 4 hours. The device is then peeled from the master and holes are punched using a 0.75mm biopsy coring needle. After that, the device is bonded to a glass slide following oxygen plasma treatment. To make the device channels hydrophobic, Aquapel is flushed into the channels, after which the device is baked in an oven for 20 mins at 65^o^C. For all the devices (DEP and flow focusing) used in this paper, the thickness of the photoresist was maintained at 25μm while the channel widths at the flow-focusing junctions were 20μm.

### Bacterial strain construction and growth

The parental wild type strain is BW25113 [35]. The entire lpoA ORF was deleted and replaced with a sacB-cat cassette using lambda Red recombinase-mediated allelic exchange (33). The Red recombinase was expressed using plasmid pKD46. The sacB-cat cassette was generated by PCR using plasmid pDS132 [36] as template and primers ANG188 and ANG189. Transformants were selected on LB Cam10 and verified by diagnostic PCR.

Next, the mutant lpoA allele was generated by two-step overlap-extension PCR. The first-round PCR products were generated using primers ANG065 and AG4 together with AG3 and ANG066, with BW25113 genomic DNA as template. The PCR products were treated with DpnI and gel-purified to get rid of the initial template DNA. The final PCR product was generated using primers ANG065 and ANG066, with the first-round PCR products as template (present in equimolar amounts). This PCR product was used to replace the sacB-cat cassette as above, with selection on LB 0% NaCl 7% (w/v) sucrose. The sacB gene confers sucrose sensitivity, allowing counterselection. Transformants were screened for chloramphenicol sensitivity (indicating loss of cassette) and verified by diagnostic PCR and sequencing. The strain produced is the LpoA K168A E. coli.

A ΔtolA::kan insertion was introduced into wild type strain BW25113 by sequential P1 transductions, with selection on LB with 10 mM sodium citrate and ampicillin at 50 μgml^-1^, and LB with 10 mM Na citrate and kanamycin at 30 μgml^-1^, respectively. The ΔtolA::kan allele is from the Keio collection E. coli gene knock-out library [35]. The strain produced has TolA knocked out but with a wild-type copy of the BW25113 LpoA gene.

The bacteria are grown in 2% Luria-Bertani (LB) broth at 37^o^C for around 10 hours. The bacterial cultures are then assayed for their optical density (OD) via spectrophotometrical measurement of absorption at 600nm. The correlation between OD and bacterial number is taken to be that 1 OD is equivalent to 5 x 10^8^ bacteria.

### Primer sequences for the construction of mutant bacterial strains

The primer sequences used for the construction of the bacterial strains are as follows: ANG188 5’-TGCCGATTTAATATTGAGCATTGCGTAAAAAAAATATCACTGGATACATTGCCCGTAGTCTGCAAATCC-3’ (50 bp upstream of lpoA and forward sacB-cat cassette primer; the 50 bp upstream of lpoA allows homologous recombination to replace the gene), ANG189 5’-CAGCCAGCGACGCGCTTGTGCTTCCCACGCATCGCCGGTCTGTTTGGTGGCCATGACCCGGGAATTACG-3’ (50 bp downstream of lpoA (reverse-complement) and rev sacB-cat cassette primer), ANG065 5’-CGCAAACAACCGGGCATTAATC-3’ (forward upstream lpoA primer, anneals 256 bp upstream of lpoA), ANG066 5’-TTTGCTGCGGGTCACACTG-3’ (reverse downstream lpoA primer, anneals 209 bp downstream of lpoA), AG3 5’-gctgcttggcgcgGCagaaaaacagcag-3’ (forward lpoA(K168A) mutagenesis primer; upper-case letters represent changes for lpoA(K168A) point mutation), AG4 5’-ctgctgtttttctGCcgcgccaagcagc-3’ (reverse lpoA(K168A) mutagenesis primer).

### Encapsulation of bacteria in monodisperse droplets

Before mixing bacteria together with the other components of the reaction, the bacterial suspension is washed 3 times by centrifugation at 3000 rpm (Eppendorf) followed by resuspension of the pellet in distilled water. The bacteria is mixed together with primers, Taqman probe and PCR mix (2X ddPCR MasterMix, Biorad). The primers and Taqman probe are used at a working concentration of 1μM and 250nM respectively. This mix is loaded into a 1 ml syringe back-filled with HFE-7500 oil, which was connected to a coaxial flow-focus device. The oil used for the carrier phase was the droplet generation oil for probes (Bio-rad). The oil flow rate is run at 400 μlhr^-1^ while the aqueous flow rate is set at 200 μlhr^-1^. The total handling time for bacteria is ∼20 minutes. The emulsion is collected into PCR tubes and thermocycled on a T100 thermocycler (Bio-Rad), with the following conditions: 10 min at 95^o^C, 35 cycles of 10 s at 95^o^C, 15 s at 55^o^C and 30 s at 70^o^C. To verify that the PCR reactions were specific, both bacterial samples were electrophoresed on a 2% agarose gel. No non-specific product was observed after imaging.

### Primer and probe sequences for Taqman PCR, LpoA amplification and sequencing

The primers for the detection of TolA are: TolA Forward 5’-GTTGATTCAGGTGCGGTAGTT-3’, TolA Reverse 5’- GCCTGCTGTTCCTTCATCTT-3’. The TolA probe sequence is 5’- /6-FAM/ATCAAACCT/ZEN/GAAGGTGGCGATCCC /3IABkFQ/-3’. The primers for LpoA amplification are: LpoA Forward 5’- TTTACTGCGCGCGTTAATTG-3’, LpoA Reverse 5’- TTGCGGCTGAGGTTGTT-3’. The primer for TOPO Vector sequencing is: M13 Forward (-20) 5’-GTAAAACGACGGCCAG-3’. All probes and primers are from IDTDNA Technologies.

### DEP sorting

Thermocycled drops are collected into a syringe filled with HFE-7500 (3M) fluorinated oil, and are left to cream for 10 minutes before starting the syringe pump. The drops are then reinjected into the DEP device at a flow rate of 50 μlhr^-1^, with the spacer oil flow rate set at 1000 μlhr^-1^. The flow rate for the 2^nd^ oil spacer at the sorting junction is set at 100 μlhr^-1^. All the oil used for spacing droplets is HFE-7500. The moat is filled with 2M NaCl salt solution, as are the salt electrodes. The PMTs (Thorlabs, PMM01 model) are connected to a computer with LABVIEW software and a FPGA data acquisition card (National Instruments) for droplet fluorescence intensity recording and electrode activation. Custom LABVIEW software is written to enable dynamic adjustments of PMT gain,droplet fluorescence intensity thresholds for sorting, electrode AC voltage pulse frequency and magnitude. The data acquisition rate for this system is 200 kHz.

### LpoA sequencing verification

Droplets from the positive DEP sort are collected into 1.5ml Eppendorf tubes. Chloroform (Sigma-Aldrich) and distilled water are pipetted over the oil, with 20μL of water used for every 200μL of chloroform and 200μL of oil. The droplets are then vortexed for 10 minutes on a shaker, and then centrifuged at 14,000rpm. The top layer of immiscible water is then extracted, of which 9μl is used for PCR amplification. The PCR amplification mixture consists of 1μM forward and reverse LpoA sequencing primers, 1X Toptaq PCR master mix (Qiagen), and template from the broken drops in a total volume of 20μL. The mixture is then thermocycled with the following conditions: 10 min at 95^o^C, 35 cycles of 10 s at 95^o^C, 15 s at 55^o^C and 30 s at 72^o^C. The PCR product is then cloned into a pCR4-TOPO vector (Life Technologies) using a TOPO TA cloning kit for sequencing (Life Technologies), as following the manufacturer’s instructions. This is transformed into electrocompetent E. coli TOP10 bacteria and streaked onto LB plates with 50μgml^-1^ kanamycin for growth at 37^o^C overnight. Colonies are picked at random for overnight growth in LB with 50μgml^-1^ kanamycin at 37^o^C, DNA extracted using a Qiagen miniprep kit, and then sent for Sanger sequencing (Quintara Biosciences). The primer used for sequencing is the M13 Forward (-20) primer.

## Supporting Information

S1 Fig Droplet size distribution. The single emulsion droplet diameters was quantified using ImageJ, with a total of 1200 drops measured for all concentrations of bacteria. The average drop volume was calculated to be 34.7 pL.
(PDF)
