# Supplementary figures and images for: PCR-Activated Cell Sorting for Cultivation-Free Enrichment and Sequencing of Rare Microbes
Source: PLoS One. 2015 Jan 28;10(1):e0113549. doi: 10.1371/journal.pone.0113549 (PMC4309575; doi:10.1371/journal.pone.0113549)

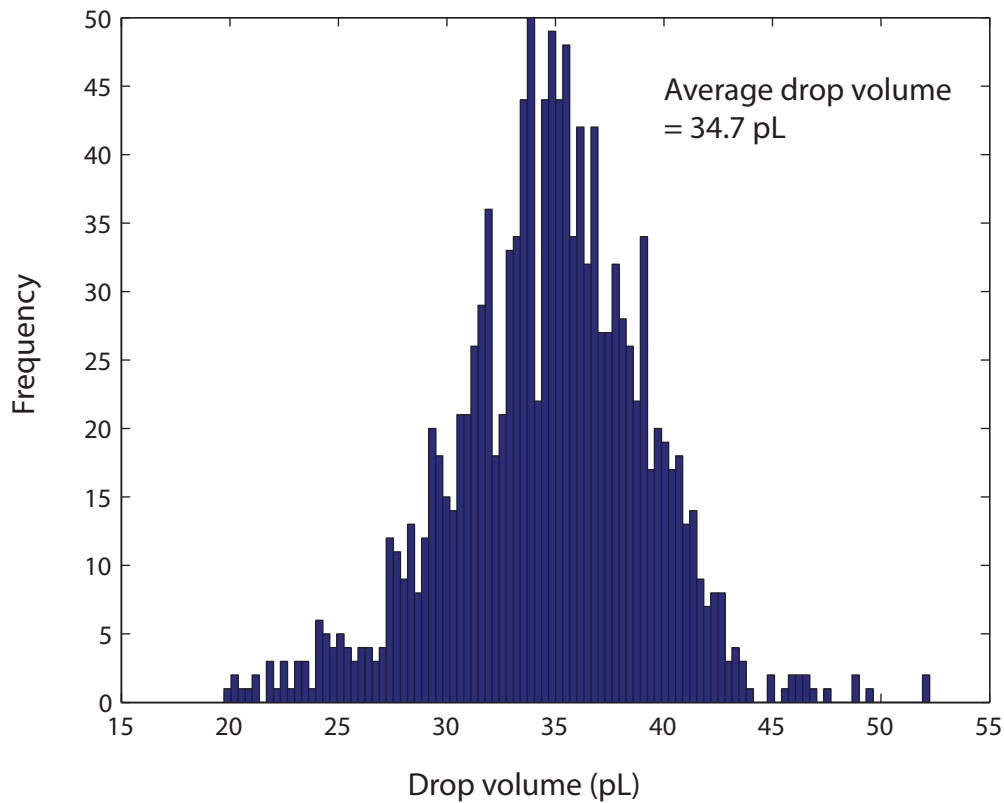

Supplement: S1 Fig — The single emulsion droplet diameters was quantified using ImageJ, with a total of 1200 drops measured for all concentrations of bacteria. The average drop volume was calculated to be 34.7 pL. (PDF) [file pone.0113549.s001.pdf]
